# Effects of early rehabilitation with a physiatrist and a registered therapist operating acute rehabilitation in mechanically ventilated ICU patients: a prospective observational study

**DOI:** 10.3389/fresc.2026.1769102

**Published:** 2026-05-01

**Authors:** Masato Ise, Yasuhisa Fujita, Satoki Furuta, Mari Kakita, Yasunori Umemoto, Shigeaki Inoue, Masanori Hamada, Fumihiro Tajima, Ken Kouda

**Affiliations:** 1 Department of Rehabilitation Medicine, School of Medicine, Wakayama Medical University, Wakayama, Japan; 2 Department of Rehabilitation Medicine, Chuzan Hospital, Okinawa, Japan; 3 Department of Rehabilitation Medicine, Graduate School of Medical Science, Kyoto Prefectural University of Medicine, Kyoto, Japan; 4 Department of Rehabilitation Medicine, Sapporo Medical University, Sapporo, Japan; 5 Department of Emergency and Critical Care Medicine, Wakayama Medical University, Wakayama, Japan; 6 Department of Rehabilitation Medicine, Okayama University Hospital, Okayama, Japan

**Keywords:** early mobilization, intensive care unit, mechanical ventilation, physiatrist, neurological responsiveness

## Abstract

**Introduction:**

Prolonged immobilization in the intensive care unit (ICU) leads to ICU-acquired weakness (ICU-AW), which delays ventilator liberation and worsens outcomes. Although early mobilization is beneficial, and the Physiatrist and Registered Therapist Operating Acute Rehabilitation (PROr) program has been associated with improved functional recovery and higher home-discharge rates in stroke populations, its effectiveness in mechanically ventilated ICU patients remains unclear. This study aimed to evaluate the safety and effectiveness of the PROr in mechanically ventilated ICU patients.

**Methods:**

We conducted a single-center, prospective observational study from 2013 to 2017. Adults requiring mechanical ventilation with a pre-admission Barthel Index (BI) ≥ 70 were included. Patients were assigned to early mobilization (EM; within 48 h of ICU admission) or usual mobilization (UM; after 48 h). Primary outcomes were duration of mechanical ventilation and ICU length of stay. Secondary outcomes included neurological responsiveness and functional recovery at discharge.

**Results:**

Sixty-nine patients were analyzed (44 EM, 25 UM), with comparable baseline characteristics. EM was associated with shorter median ventilation duration (5.5 vs. 7.7 days; *p* < 0.05) and ICU stay (9.1 vs. 11.6 days; *p* < 0.05). Eye-opening scores on the Glasgow Coma Scale significantly improved during mobilization in both groups, whereas Motor-response scores showed no significant differences. Functional outcomes at discharge (BI, FIM) were similar between groups. No adverse events occurred during mobilization.

**Conclusions:**

Mobilization within 48 h under the PROr did not induce any clinical adverse events and significantly reduced ventilation duration and the length of ICU stay. Mobilization of mechanically ventilated unconsciousness ICU patients may improve levels of consciousness.

## 1 Introduction

Prolonged immobilization in critically ill patients is a major contributor to intensive care unit–acquired weakness (ICU-AW) (1, 2). It delays liberation from mechanical ventilation and contributes to persistent functional disability (1, 2). Over the past decade, early mobilization (EM) has become an integral component of critical care. Randomized controlled trials and meta-analyses have demonstrated improvements in muscle strength and reductions in delirium, with some studies also reporting shorter ICU length of stay (3–6). A landmark trial of goal-directed mobilization in a surgical ICU reported both a shorter ICU length of stay and greater functional independence at hospital discharge (7). Systematic reviews and meta-analyses suggest that mobilization within 24 to 48 h is associated with the most favorable outcomes (8–10). More recent studies have linked early activity to better long-term functional and cognitive outcomes (11–13). Progressive early rehabilitation protocols have also been shown to be feasible in mechanically ventilated ICU patients (13). In many of these studies, mobilization was performed by physical therapists or nurses as part of structured protocols (3–7). The role of physicians was usually limited to granting medical clearance or addressing contra-indications, without taking a direct role in leading mobilization. In contrast, at our institution, EM is delivered through the PROr (Physiatrist and Registered Therapist Operating Acute Rehabilitation) program. In this model, a physiatrist conducts an immediate bedside assessment before rehabilitation begins and collaborates with a registered rehabilitation therapist to initiate mobilization as soon as the patient is hemodynamically stable, regardless of consciousness level or depth of sedation. The physiatrist prescribes individualized mobilization plans and conducts sessions together with the rehabilitation therapist. The physiatrist also works in close coordination with intensivists to ensure continuous medical management. This collaborative involvement of physiatrists and registered therapists may help overcome the limitations of prior approaches and support the efficient delivery of EM. In stroke populations, the PROr has been associated with higher home discharge rates and greater functional recovery when mobilization is initiated within 24 h of onset (14, 15). Its effectiveness in mechanically ventilated ICU patients has not yet been established. We conducted a prospective observational study to evaluate the effect of the PROr in mechanically ventilated ICU patients. We compared patients who initiated mobilization within 48 h of mechanical ventilation EM with those who began after 48 h (usual mobi-lization, UM). We hypothesized that EM would be associated with shorter durations of mechanical ventilation and ICU length of stay. We also expected EM to result in improved functional recovery at ICU and hospital discharge without increasing adverse events.

## 2 Method

### 2.1 Patients and study design

We conducted a prospective observational study of adult patients (≥18 years) ad-mitted to the intensive care unit (ICU) of our hospital who required mechanical ventilation between January 1, 2013 and December 31, 2017. Eligibility required a Barthel Index (BI) score ≥70 within 2 weeks prior to hospital admission.

**Figure 1:**
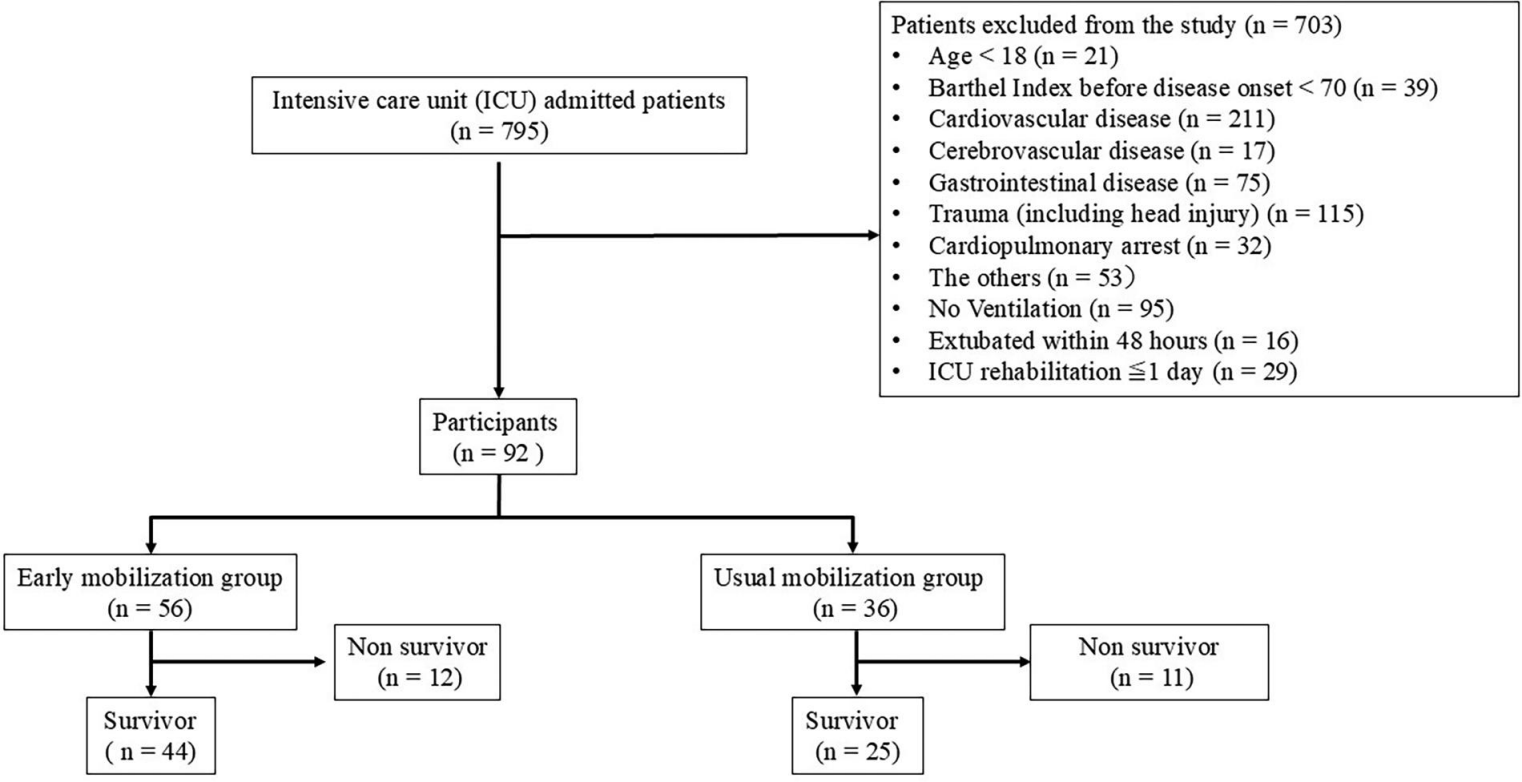
Flow diagram of patient selection.

Exclusion criteria included acute neuromuscular disorders; elevated intracranial pressure; limb amputation; pathological fractures; spinal instability; postoperative status after gastrointestinal or cardiovascular surgery; receipt of ≤1 day of ICU rehabilitation; or clinical deterioration during hospitalization, including in-hospital death. The flow of patient enrollment, exclusion, and group allocation is shown in Figure 1. The EM group comprised patients admitted on weekdays who initiated mobilization within 48 h of mechanical ventilation onset, whereas the UM group included patients admitted during weekends or holidays who began mobilization after 48 h. Although a 72-h threshold has been commonly used in previous studies (4), we defined 48 h as the cutoff to reflect our institutional practice. Because rehabilitation services were not available on weekends or holidays at our hospital during the study period, group assignment was not based on randomization or predefined clinical criteria, but rather on the actual timing of mobilization initiation in routine clinical practice. At our hospital, it is standard clinical practice for acute care specialists to request a physiatrist consultation immediately after ICU admission of critically ill patients Based on a comprehensive clinical assessment, mobilization strategies are initiated according to the patient's hemodynamic status, regardless of consciousness level or depth of sedation. This approach is implemented through the PROr program established at our hospital.

### 2.2 Intervention procedures

At our hospital, ICU rehabilitation was delivered through the PROr program. After ICU admission, the intensivist consulted a physiatrist, who performed a bedside assessment. Based on respiratory and hemodynamic stability, the physiatrist and registered therapist jointly determined the appropriate mobilization level for each patient. Mobilization was initiated regardless of consciousness level or sedation depth, provided that the patient was considered clinically stable. The mobilization level was reassessed daily and progressed stepwise as tolerated. If any predefined termination criteria were met during the session, the intervention was interrupted. Rehabilitation continued until ICU discharge and after ICU discharge. Mobilization was performed according to a four-level protocol based on respiratory and hemodynamic stability and level of consciousness.

Level 1 was indicated for unconscious patients with a Richmond Agitation-Sedation Scale (RASS) score of −5 and unstable respiratory or circulatory status, as judged by the intensivist and a physiatrist. At this level, nurses performed passive range-of-motion exercises of the upper and lower extremities once daily, with at least five repetitions per joint. These exercises included shoulder flexion, elbow flexion and extension, wrist palmar flexion and dorsiflexion, finger flexion and extension, hip and knee flexion and extension, and ankle plantar flexion and dorsiflexion. Registered therapists additionally provided 20 min of contracture prevention and respiratory rehabilitation.

Level 2 was indicated for patients receiving mechanical ventilation via endotracheal intubation or tracheostomy, with a RASS score between −5 and −3 and stable respiratory and circulatory status, as determined by the intensivist and a physiatrist. At this level, nurses performed passive range-of-motion exercises once daily and head-up positioning for 20 min twice daily. Registered therapists conducted sitting at the edge of the bed for 20 min twice daily in addition to passive limb exercises and respiratory rehabilitation.

Level 3 was indicated for mechanically ventilated patients with a RASS score between −2 and +1. At this level, nurses performed passive range-of-motion exercises and limb exercises once daily and assisted wheelchair sitting or head-up positioning for 20 min twice daily. Registered therapists provided sitting at the edge of the bed or supported standing for 20 min twice daily, together with active or active-assisted limb exercises.

Level 4 was indicated for patients who were able to maintain standing with minimal assistance, as determined by registered therapists. At this level, nurses performed passive range-of-motion exercises once daily and assisted transfer to a wheelchair for 20 min twice daily. Registered therapists provided stepping or gait training for 20 min twice daily. If feasible, patients were also instructed to perform self-exercise of the upper and lower extremities, consisting of 10 repetitions of limb elevation exercises three times per day.

For eligible patients, registered therapists intervened separately according to the patient's condition. During mobilization and out-of-bed activities, registered therapists and nurses jointly monitored patient safety. When necessary, rehabilitation was performed in the presence of the intensivist and/or physiatrist.

Termination criteria for rehabilitation were as follows: mean arterial pressure <60 or >110 mmHg; systolic blood pressure ≥200 mmHg; heart rate <40 or >130 beats/min; respiratory rate <5 or >40 breaths/min; oxygen saturation ≤88%; increased intracranial pressure; active gastrointestinal bleeding; acute coronary syndrome; intermittent hemodialysis (excluding continuous renal replacement therapy); agitation requiring an increase in sedative medication; airway instability; and new initiation or dose escalation of vasopressors or antiarrhythmic agents.

In contrast to usual care reported in prior ICU rehabilitation studies, which often included passive range-of-motion exercises or physical therapy delivered only when ordered (3), the PROr program prescribed physiatrist assessment of neurological, cardiovascular, respiratory, and musculoskeletal status to guide progression to higher levels of mobilization when clinically appropriate.

### 2.3 Ethics statement

This study was conducted in accordance with the Declaration of Helsinki and approved by the Ethics Committee of Wakayama Medical University Hospital (approval number: 1194; approval date: 30 January 2013). All eligible patients or, when applicable, their legally authorized representatives, were informed orally and in writing about the purpose and procedures of the study by investigators who were not directly involved in their clinical care to minimize coercion. Written informed consent was obtained from each participant or their representative before enrollment. All signed consent forms were securely stored under the supervision of the principal investigator.

### 2.4 Outcome measures

All outcome measures were assessed by personnel blinded to group allocation. Severity of illness at ICU admission was assessed using the Acute Physiology and Chronic Health Evaluation II (APACHE II) and Sequential Organ Failure Assessment (SOFA) scores. APACHE II incorporates acute physiological variables, age, and chronic health status, yielding a total score from 0 to 71, with higher scores indicating greater severity and mortality risk. SOFA evaluates dysfunction across six organ systems (respiratory, cardiovascular, hepatic, coagulation, renal, and neurological), with total scores ranging from 0 to 24. The Richmond Agitation-Sedation Scale (RASS), a 10-point scale ranging from +4 (combative) to −5 (unarousable), was used to assess sedation and agitation. Functional outcomes were measured using the Barthel Index (BI) and the Functional Independence Measure (FIM). The BI evaluates independence in basic activities of daily living with scores ranging from 0 to 100, while the FIM assesses motor and cognitive function across 18 items scored on a 7-point scale, with total scores ranging from 18 to 126. BI and FIM scores were recorded at ICU admission, ICU transfer, and hospital discharge. Changes (Δ) in BI and FIM from ICU admission to transfer and discharge were also calculated. Adverse events were recorded according to the National Coordinating Council for Medication Error Reporting and Prevention index (16) and at our hospital, all staff are required to report an Adverse event (AE) to their corresponding risk manager.

An AE during rehabilitation means any instance that has caused or may have caused further physical or psychological injury to the patient (17).

### 2.5 Statistical analysis

Continuous variables are presented as mean ± standard deviation (SD) or median (interquartile range, IQR), as appropriate. The Mann–Whitney U test was used to compare continuous variables between groups, and categorical variables were analyzed using the chi-square test. All statistical analyses were performed using *BellCurve for Excel* (Social Survey Research Information Co., Ltd., Tokyo, Japan). A two-sided *p* value of <0.05 was considered statistically significant.

## 3 Result

### 3.1 Participant characteristics

Table 1 presents the characteristics of the study population, which included 44 patients in the EM group (mobilization within 48 h of ICU admission) and 25 in the UM group (mobilization after 48 h). There were no significant differences between the EM and UM groups in age, sex, disease severity at ICU admission (as measured by APACHE II and SOFA scores), level of consciousness (RASS), or baseline functional status (BI and FIM). No clinical adverse events occurred during and after mobilization in either the EM or UM group. The time from ICU admission to rehabilitation consultation was significantly shorter in the EM group compared with the UM group. There was also no significant difference in in-hospital mortality between the two groups.

**Table 1:** Participants characteristics.

Characteristic	EM group (*n* = 44)		UM group (*n* = 25)		*P*-Value
	Mean ± SD	Median (IQR)	Mean ± SD	Median (IQR)	
Age (years)	66.2 ± 16.5	70 (60–77)	64.8 ± 15.0	68 (56–75)	0.66
Gender, male (number)	29 (66%)		18 (72%)		0.79
Time to referral (hours)	24.2 ± 12.5	22 (15–36)	90.3 ± 40.8	70 (64–100)	<0.01
APACHE II at ICU admission	22.3 ± 7.5	22 (17–28)	24.4 ± 7.8	25 (20–29)	0.28
SOFA at ICU admission	7.8 ± 3.4	8 (6–10)	8.0 ± 3.2	7 (6–11)	0.73
RASS at ICU admission	−2.6 ± −1.5	−2 (−4–−1.5)	−2.0 ± −1.5	−2 (−3–−1)	0.15
BI at ICU admission	0	0	0	0	NaN
FIM at ICU admission	19.1 ± 2.5	18 (18–18)	19.7 ± 3.1	18 (18–22)	0.66
Mortality (number)	12 (21%)		11 (30%)		0.48

Values are presented as mean ± standard deviation (SD) and median (interquartile range [IQR]).

EM group, early mobilization group; UM group, usual mobilization group; APACHE II, Acute Physiology and Chronic Health Evaluation II; SOFA, Sequential Organ Failure Assessment; RASS, Richmond Agitation–Sedation Scale; BI, Barthel Index; FIM, Functional Independence Measure.

*P*-values were calculated using the t-test or Mann–Whitney U test for continuous variables and the chi-square test for categorical variables.

### 3.2 Duration of mechanical ventilation and the length of ICU stay

**Figure 2:**
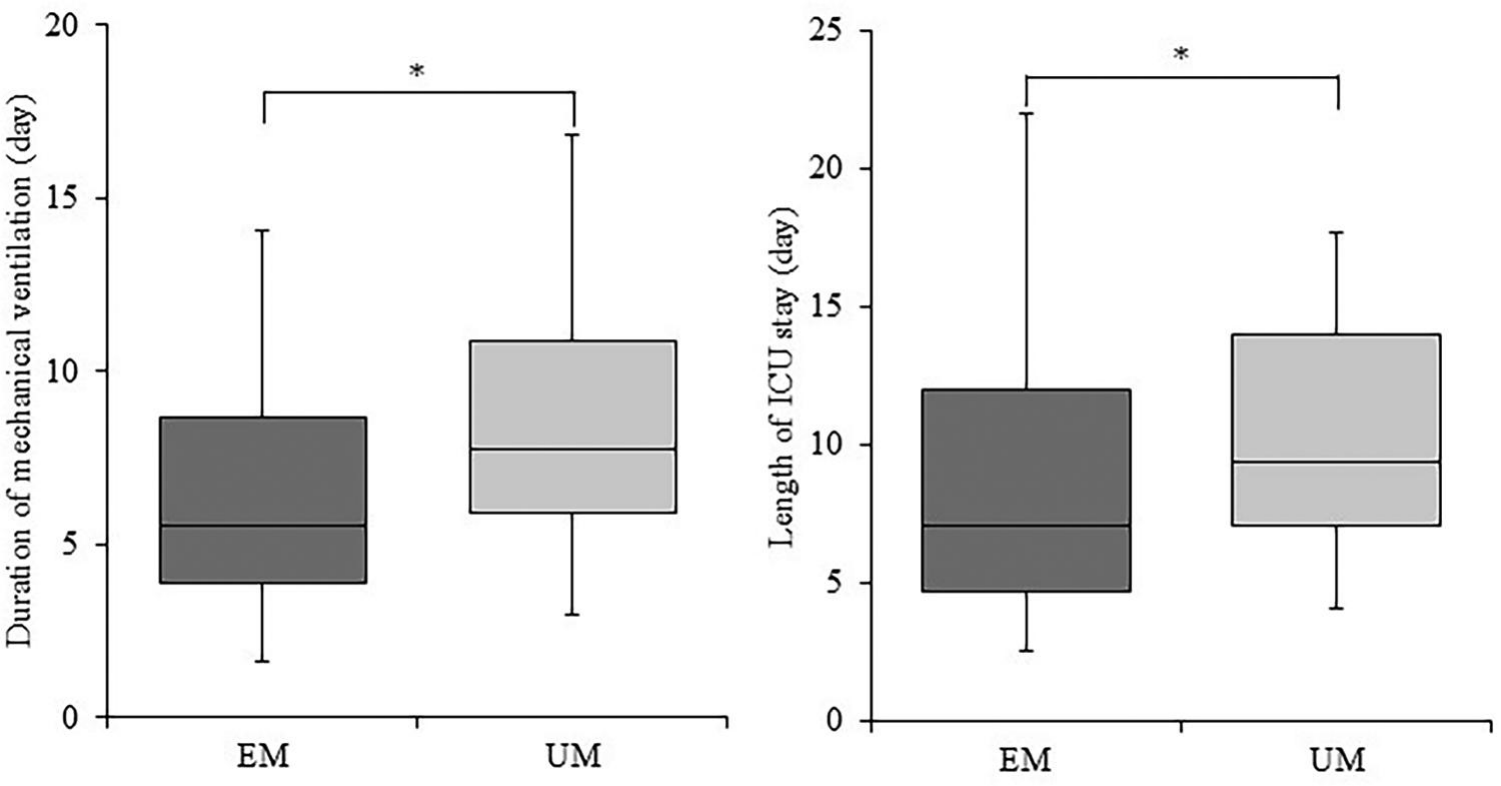
Duration of mechanical ventilation and length of ICU stay in the early mobilization (EM) and usual mobilization (UM) groups. Box-and-whisker plots show the duration of mechanical ventilation (left) and length of ICU stay (right) for the early mobilization (EM) and usual mobilization (UM) groups. Boxes represent the interquartile range (IQR), horizontal lines indicate the median, and whiskers show the range. *P < 0.05. ICU, intensive care unit.

As shown in Figure 2, the EM group had a significantly shorter duration of mechanical ventilation compared with the UM group, with a median reduction of 1.7 days (5.5 vs. 7.7 days, *P* < 0.05). Similarly, ICU length of stay was significantly shorter in the EM group by approximately 2.6 days (9.1 vs. 11.6 days, *P* < 0.05). Table 2 shows that the period from the first physiatrist consultation to extubation did not differ significantly between the EM and UM groups.

**Table 2:** Duration from physiatrist assessment to extubation in the EM and UM groups, presented as median (IQR) and mean ± SD.

Variable	EM group (*n* = 44)		UM group (*n* = 25)		*P*-Value
	Mean ± SD	Median (IQR)	Mean ± SD	Median (IQR)	
Duration from physical examination and diagnosis of physiatrists to extubation	6.9 ± 6.9	4.6 (3.0–7.6)	5.8 ± 6.2	3.4 (2.4–7.5)	0.40

Values are expressed as mean ± standard deviation (SD) and median (interquartile range, IQR). EM, early mobilization; UM, usual mobilization.

### 3.3 SOFA and RASS scores

As shown in Table 3, there were no significant differences between the EM and UM groups in SOFA scores at ICU admission or discharge, or in the change in SOFA score (ΔSOFA). Similarly, RASS scores at ICU admission and discharge were not significantly different between groups. The change in RASS score (ΔRASS) tended to be greater in the EM group, but the difference did not reach statistical significance (*P* = 0.08).

**Table 3:** SOFA and RASS scores at ICU transfer and discharge, and their changes (ΔSOFA, ΔRASS), in the EM and UM groups. Values are median (IQR) and mean ± SD.

Variables	EM group (*n* = 44)		*P*-Value (Within-group)	UM group (*n* = 25)		*P*-Value (EM vs. UM)	*P*-Value (Within-group)
	Mean ± SD	Median (IQR)		Mean ± SD	Median (IQR)		
SOFA at ICU admission	7.8 ± 3.4	8 (6–10)	<0.001	8.0 ± 3.2	7 (6–11)	0.81	<0.001
SOFA at ICU discharge	3.5 ± 1.9	3 (2–5)		4.5 ± 3.5	4 (2–5)	0.33	
⊿SOFA	−4.2 ± 3.2	−4 (−6.3–−2.0)		−3.6 ± 3.0	−3 (−5–−2)	0.23	
RASS score at ICU admission	−2.6 ± 1.5	−2 (−4–−1.5)	<0.001	−2.0 ± 1.5	−2 (−3–−1)	0.15	<0.001
RASS score at ICU discharge	−0.3 ± 0.9	0 (−0.3–0)		−0.5 ± 1.0	0 (0–0)	0.61	
⊿RASS	2.3 ± 1.5	2 (1–4)		1.6 ± 1.1	1 (1–2)	0.08	

Values are expressed as mean ± standard deviation (SD) and median (interquartile range, IQR). ΔSOFA and ΔRASS indicate the differences between ICU admission and discharge. EM, early mobilization; UM, usual mobilization; SOFA, Sequential Organ Failure Assessment; RASS, Richmond Agitation–Sedation Scale; ICU, intensive care unit.

### 3.4 Glasgow Coma Scale (GCS) eye and motor score changes before and during mobilization

Most patients did not open their eyes before mobilization; however, we observed that active mobilization prompted voluntary eye opening. Therefore, we analyzed eye-opening and motor response scores before and during mobilization, excluding patients with M6 and E4 responses to avoid ceiling effects. Verbal response scores were not included because most patients were mechanically ventilated. In both the EM and UM groups, eye-opening scores during mobilization improved significantly compared with scores before mobilization, whereas motor response scores did not show significant improvement. Both groups demonstrated modest increases in GCS motor response scores during mobilization compared with baseline. However, within-group comparisons did not reach statistical significance in either group, and between-group differences were also not significant (Table 4).

**Table 4:** GCS scores before and during mobilization in the EM and UM groups (E4 or M6 excluded).

GCS component	Group	Before mobilization		During mobilization		*P*-Value (Within-group)	*P*-Value (EM vs. UM)
		Mean ± SD	Median (IQR)	Mean ± SD	Median (IQR)		
GCS Eye opening score	EM group	2.7 ± 0.7	3 (3–3)	3.2 ± 0.9	3 (3–4)	<0.01	0.12
	(*n* = 29)						
	UM group	2.2 ± 1.0	3 (1–3)	2.9 ± 1.2	3 (2.5–4)	<0.05	0.57
	(*n* = 15)						
GCS Motor response score	EM group	3.1 ± 1.9	4 (1–5)	3.6 ± 2.3	5 (1–4)	0.34	0.41
	(*n* = 15)						
	UM group	2.7 ± 1.7	3 (1–4)	3.0 ± 1.6	3 (2–4)	0.58	0.44
	(*n* = 9)						

Values are expressed as mean ± standard deviation (SD) and median (interquartile range, IQR). EM, early mobilization; UM, usual mobilization; GCS, Glasgow Coma Scale.

### 3.5 Functional outcomes (Barthel Index and functional independence measure)

There were no significant differences between the EM and UM groups in BI or FIM scores at ICU discharge or hospital discharge. Similarly, no significant differences were observed in the changes in BI and FIM scores (ΔBI and ΔFIM) during hospitalization between the two groups (Table 5).

**Table 5:** BI and FIM scores and their changes at ICU and hospital discharge in the EM and UM groups. Values are mean ± SD.

BI	EM group (*n* = 44)	*P*-Value (vs. admission)	UM group (*n* = 25)	*P*-Value (vs. admission)
	Mean ± SD		Mean ± SD	
ICU admission	0		0	
ICU discharge	12.8 ± 15.0	<0.01	17.6 ± 26.0	<0.01
Hospital discharge	64.2 ± 32.4	<0.01	52.8 ± 32,4	<0.01
FIM				
ICU admission	19.1 ± 2.5		19.8 ± 3.1	
ICU discharge	40.5 ± 19.2	<0.01	41.7 ± 22.3	<0.01
Hospital discharge	89.0 ± 34.8	<0.01	83.0 ± 34.7	<0.01

Values are expressed as mean ± standard deviation (SD). EM, early mobilization; UM, usual mobilization; BI, Barthel Index; FIM, Functional Independence Measure; ICU, intensive care unit.

## 4 Discussion

### 4.1 Summary of key findings

The present study demonstrated the following findings:

(1) patients who initiated mobilization within 48 h had shorter duration of mechanical ventilation and ICU stay than those who started after 48 h; (2) eye-opening scores during mobilization increased in both the EM and UM groups; and (3) no significant between-group differences were observed in discharge BI or FIM outcomes. These results suggest that earlier initiation of mobilization may be associated with shorter duration of mechanical ventilation and ICU stay. In addition, mobilization was associated with improved eye-opening responses during rehabilitation in both groups.

However, because all enrolled patients underwent rehabilitation and no usual care group without mobilization was included, this study cannot determine whether mobilization itself is effective compared with usual care alone. The most notable finding of this study was that ICU mobilization significantly improved eye-opening scores in both groups, suggesting a meaningful tool to enhance consciousness status in ICU patients. Early mobilization may represent an effective method to promote arousal in patients with impaired consciousness.

### 4.2 Neurological improvement during mobilization

Improvement in impaired consciousness during mobilization in ICU patients may be explained by several physiological mechanisms. A previous study reported that transitioning from the supine to sitting position significantly increased Glasgow Coma Scale (GCS) scores in patients with cerebral disorders (18). The authors suggested that upright posture imposes anti-gravity loading on the trunk and lower limbs, which leads to sustained activation of anti-gravity muscles and increased proprioceptive and tactile input from the lower extremities, trunk, and buttocks. These afferent signals are transmitted to the central nervous system, where they may stimulate the ascending reticular activating system to promote arousal. A previous physiological study demonstrated that in the seated position, vertebral artery (VA) blood flow is maintained despite a significant reduction in internal carotid artery flow (19). During upright posture, venous drainage shifts from the internal jugular vein to the vertebral veins. The VA supplies posterior circulation, including the brainstem and reticular activating system. Preserved VA flow in the upright position may help sustain posterior cerebral perfusion and support arousal in patients with impaired consciousness during mobilization. Mobilization can also improve pulmonary mechanics and oxygenation (4, 7). It may optimize cerebral perfusion and increase exposure to environmental stimulation. These combined effects may enhance wakefulness. In this study, we directly and prospectively evaluated changes in GCS during mobilization. Our findings provide objective evidence of transient neurologic benefits in critically ill patients. Although motor response scores did not change significantly, the improvement in eye-opening scores was clinically meaningful.

### 4.3 Absence of between-group differences in functional outcomes

Despite differences in mobilization initiation time, no significant differences were observed between the EM and UM groups in BI or FIM scores at ICU or hospital discharge. However, this finding should be interpreted cautiously. One possible explanation is that assessment at ICU or hospital discharge may have been too early to detect meaningful differences in longer-term functional recovery. Therefore, follow-up beyond discharge may provide more informative data regarding the true impact of early mobilization on recovery trajectory and activities of daily living.

The absence of differences in final functional outcomes may reflect both the physiological mechanisms of deconditioning during critical illness and the therapeutic effects of rehabilitation. Prolonged immobilization in critically ill patients rapidly induces skeletal muscle atrophy and weakness, driven by accelerated muscle protein catabolism, altered neuromuscular junction transmission, and mitochondrial dysfunction. Measurable declines can occur within the first week of ICU admission (20, 21). Cardiovascular deconditioning also develops, characterized by reduced cardiac output, orthostatic intolerance, and impaired autonomic regulation, all which limit exercise tolerance and delay mobility recovery (22). Early mobilization may attenuate such deconditioning, contributing to the shorter duration of mechanical ventilation and ICU stay observed in the EM group compared with the UM group.

In our study, both groups received intensive rehabilitation through PROr program from ICU admission until hospital discharge. Our institution has a structured rehabilitation system involving close collaboration among intensivists, physiatrists, nurses, and registered therapists, which may not be readily available in all hospitals. Therefore, the applicability of our findings to healthcare settings with fewer rehabilitation resources or different organizational systems should be interpreted cautiously. Moreover, after ICU discharge, all patients underwent daily bedside rounds by physiatrists and rehabilitation therapists, followed by weekday mobilization sessions lasting min to the maximum extent tolerated. This standardized post-ICU rehabilitation was provided to both groups under the same institutional framework. Such continuous and comparable rehabilitation may have enabled patients in the UM group to achieve substantial functional gains, allowing them to reach levels comparable to those of the EM group by discharge. Once mobilization begins, both early and later initiation groups may undergo similar neuromuscular and cardiopulmonary adaptations in response to training (23). Such adaptations may partly explain the convergence in functional outcomes at discharge. This interpretation is consistent with the TEAM trial, which demonstrated that very early and intensive mobilization did not improve long-term functional outcomes at 6 months compared with usual care (24).

### 4.4 Clinical implications for ICU practice

Shortening the duration of mechanical ventilation and ICU stay has clear clinical and economic benefits. Early liberation from mechanical ventilation reduces the risks of ventilator-associated pneumonia, sedation-related complications, and ICU-acquired weakness (20, 25). Similarly, a reduced ICU length of stay lowers the incidence of nosocomial infections and optimizes healthcare resource utilization (7). These advantages support the integration of early rehabilitation into routine ICU care for eligible patients.

### 4.5 Recommendations and future directions

In this study, no adverse events related to mobilization occurred in either group. This finding confirms that early rehabilitation can be safely delivered even in mechanically ventilated patients. Our results suggest that PROr, involving physiatrists working in collaboration with registered therapists, may facilitate the safe and effective implementation of early mobilization. This contrasts with previous studies that did not include dedicated physiatrists (3–7). Our findings support existing evidence that initiating rehabilitation within 48 h of ICU admission may be beneficial whenever feasible. Future multicenter trials are warranted to clarify the independent effects of mobilization timing, intensity, and physiatrist involvement on patient outcomes. Such studies should also evaluate the feasibility and cost-effectiveness of PROr models that incorporate physiatrist participation across diverse ICU settings.

### 4.6 Limitations

This study has certain limitations. It was conducted at a single center with dedicated rehabilitation resources and a structured multidisciplinary collaboration system, which may limit the generalizability of the findings to other healthcare settings. The study was prospective and observational without randomization, introducing the possibility of selection bias and residual confounding. In addition, the unequal number of patients in the EM and UM groups represents a methodological limitation. No propensity score matching or multivariable adjustment was performed, and unmeasured confounders may have affected both group assignment and outcomes. Finally, although the sample size was statistically sufficient for group comparison, the relatively small number of patients may have reduced the robustness of the findings and the ability to detect differences in secondary outcomes.

## 5 Conclusions

Initiation of mobilization within 48 h under the PROr program was associated with shorter durations of mechanical ventilation and ICU length of stay in mechanically ventilated ICU patients. Although no significant differences between-group were observed in functional outcomes at discharge, eye-opening scores improved significantly during mobilization in the union of either EM or UM group. These findings suggest that the PROr program, in which mobilization is initiated regardless of consciousness level or depth of sedation, may be clinically meaningful and may represent an effective rehabilitation strategy for mechanically ventilated ICU patients. Early mobilization, including sitting and standing, in unconscious patients receiving mechanical ventilation in the ICU may be an effective strategy for improving levels of consciousness.

## Data Availability

The original contributions presented in the study are included in the article/Supplementary Material, further inquiries can be directed to the corresponding author.
